# H_2_O_2_ Is Involved in the Metallothionein-Mediated Rice Tolerance to Copper and Cadmium Toxicity

**DOI:** 10.3390/ijms18102083

**Published:** 2017-10-01

**Authors:** Hongxiao Zhang, Shufang Lv, Huawei Xu, Dianyun Hou, Youjun Li, Fayuan Wang

**Affiliations:** 1 College of Agriculture, Henan University of Science and Technology, Luoyang 471023, China; zhanghx21@126.com (H.Z.); lvshufang780515@sina.com (S.L.); xhwcyn@163.com (H.X.); dianyun518@163.com (D.H.); 2 College of Environment and Safety Engineering, Qingdao University of Science and Technology, Qingdao 266042, China

**Keywords:** H_2_O_2_, *ricMT*, rice suspension cells, Cu stress, Cd stress

## Abstract

Cadmium (Cd) and excess copper (Cu) are toxic to plants, causing a wide range of deleterious effects including the formation of reactive oxygen species. Metallothioneins (MTs) may protect plant cells from heavy metal toxicity by chelating heavy metals via cysteine thiol groups. They may also function as antioxidants. The study investigated the relationship of H_2_O_2_ production and *ricMT* expression in rice radicles and rice suspension cells under Cu or Cd stress. The results showed that H_2_O_2_ production in the rice radicles increased before Cu-induced *ricMT* expression, and after Cd-induced *ricMT* expression. Rice suspension cells of sense- and antisense-*ricMT* transgenic lines were obtained by an *Agrobacterium*-mediated transformation. Overexpression of *ricMT* significantly decreased the death rate of rice cells, which was accompanied by blocked H_2_O_2_ accumulation in rice suspension cells subject to Cu and Cd stress. Our findings confirm that H_2_O_2_ is involved in the *MT*-mediated tolerance of Cu and Cd toxicity in rice.

## 1. Introduction

Copper (Cu) is essential for plant growth and development. However, excessive levels of essential and non-essential metals, including cadmium (Cd), are toxic to plants, with a wide range of deleterious effects [1]. As a redox-active metal, Cu can catalyze the formation of reactive oxygen species (ROS) such as superoxide anion (O_2_^•−^), hydrogen peroxide (H_2_O_2_), and hydroxyl radical (HO·) via Fenton-type reactions. The O_2_^•−^ generated is usually dismutated to H_2_O_2_ via superoxide dismutase (SOD) [2]. By contrast, Cd is not redox-sensitive and does not participate in Fenton-type reactions, however it can promote ROS production probably via the depletion of thiol compounds [3].

ROS are highly toxic and can oxidize biological macromolecules such as lipids, proteins, and nucleic acids, causing lipid peroxidation, membrane damage, and enzyme inactivation. To scavenge ROS and alleviate their deleterious effects, plants have evolved diverse protective mechanisms, including various enzymes and non-enzymatic systems, to adjust ROS levels [4]. However, ROS can serve as signaling molecules for the induction of plant responses to environmental stresses such as heavy metals [5]. Plants possess over 150 genes that encode different ROS-detoxifying or ROS-producing enzymes forming a well-organised ROS gene web [4]. Cho and Seo [6] reported that reduced H_2_O_2_ accumulation increased Cd tolerance in *Arabidopsis* seedlings. H_2_O_2_ supplied exogenously to rice seedlings increased the glutathione level and protected them against subsequent Cd stress [7]. The improved Cd tolerance in rice seedlings may be due to a stimulated antioxidant system and Cd sequestration [8]. Although many physiological and biochemical analyses have examined the responses of plants to metal toxicity, the role of H_2_O_2_ in regulating metal-responsive protein expression in plants is still not completely understood.

Metallothioneins (MTs) are a class of low molecular weight, cysteine (Cys)-rich, metal-binding proteins. In animals, MTs are involved in maintaining the homeostasis of essential metals and metal detoxification, and have been implicated in a range of other physiological processes, including ROS scavenging and regulating cell growth and proliferation [9]. Plant MTs may protect cells against the toxic effects of heavy metals by chelating them via Cys thiol groups, and they are also proposed to function as antioxidants [3,10]. Note that plant MTs are induced by a variety of environmental stimuli including peroxides, drought, cold, salt, and heavy metal toxicity, and these stimuli are accompanied by the production of ROS [11,12,13,14,15]. Consequently, the increased MT expression in stressed plants may be important for ROS scavenging or signaling [16,17]. Although studies have attempted to determine the functional action of MTs in plants [12,15,18,19,20], additional information of linking MTs to ROS in the response to heavy metal stress in plants is still needed.

Rice possesses more *MT* genes than other plant species that have been studied. The MT isoforms expressed in rice are classified into four types based on their Cys content and the organization of the Cys residues at their N- and C-termini [21]. Some rice MTs have been shown to be ROS scavengers [20,22]. In our previous study, proteomic evidence showed that a MT-like protein, called ricMT by Yu et al. [23] and OsMT2c or OsMT-I-2b by Zhou et al. [21], and a copper/zinc superoxide dismutase (CuZn-SOD) are Cu-responsive proteins in germinating rice seeds [24,25,26], and OsMT2c transcription was also induced in response to both Cu and H_2_O_2_ [18], which suggests that H_2_O_2_ and MTs are connected in rice under metal stress. To clarify the relationship between MTs and H_2_O_2_ in rice under Cu and Cd stress, we investigated the H_2_O_2_ production, ricMT and CuZn-SOD mRNA expression patterns in the radicles of germinating rice seeds under Cu and Cd stress, as well as Cd- and Cu-induced cell death and H_2_O_2_ production in rice suspension cells of the wild-type (WT), and transgenic lines overexpressing and under expressing *ricMT*.

## 2. Results

### 2.1. Effects of Cu and Cd on H_2_O_2_ Production in Rice Radicles

**Figure 1:**
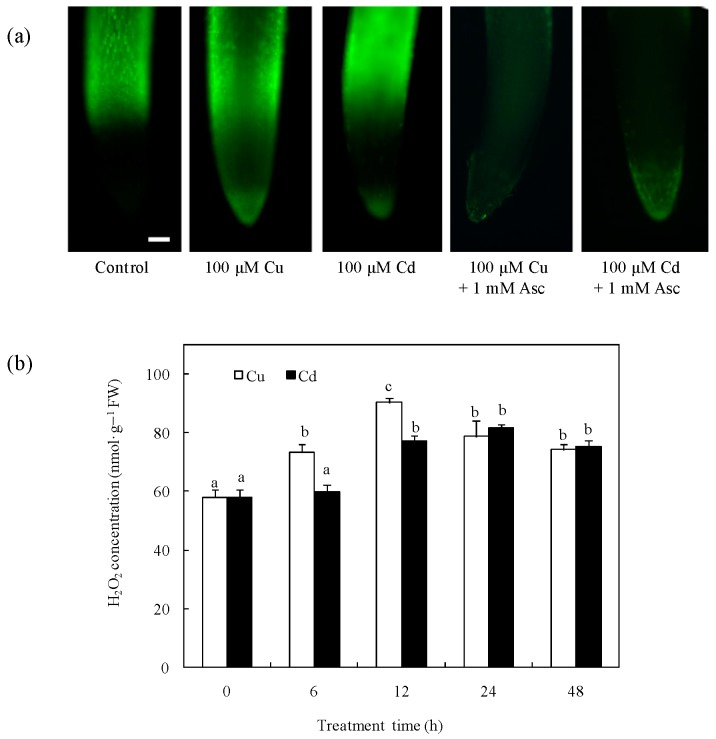
Cu- and Cd-induced H_2_O_2_ accumulation in rice radicles. (**a**) Histochemical detection of H_2_O_2_ in rice radicles under different treatment for 12 h; (**b**) The total contents of H_2_O_2_ in rice radicles under varied Cu or Cd treatment time. Germinating rice embryos were treated with 1 mM ascorbic acid (Asc) solution for 12 h, or treated with 100 µM CuSO_4_ and 100 µM CdCl_2_ solution for 0, 3, 6, 12, 24, and 48 h. Subsequently radicles from germinating rice seeds were incubated in 20 μM H_2_DCFDA for 20 min or were homogenized and the H_2_O_2_ content assayed by spectrophotometry. Bar, 100 μm. Experiments were repeated at least three times with similar results. Values are means ± SE (*n* = 3) of three separate experiments. Means denoted by the same letter did not significantly differ at *p* < 0.05 according to Duncan’s multiple range test.

To understand the effects of Cu and Cd on H_2_O_2_ production in rice, we investigated the H_2_O_2_ production in rice radicles by 2′,7′-dichlorodihydrofluorescein diacetate (H_2_DCFDA) staining. Compared with the control and the treatment with the H_2_O_2_ scavenger, Asc, the 12-h treatments with 100 μM Cu or 100 μM Cd significantly increased the H_2_O_2_ production of radicles (Figure 1a). When the H_2_O_2_ concentrations were assayed spectrophotometrically, H_2_O_2_ gradually increased during the first 12 h of 100 µM Cu exposure and then decreased slightly but remained higher than that of the control; however, the Cd-induced H_2_O_2_ happened only after 12 h of 100 µM Cd exposure, which lagged behind that of Cu exposure (Figure 1b).

### 2.2. Cu and Cd Up-Regulate the ricMT and CuZn-SOD Gene Expression in Rice Radicles

**Figure 2:**
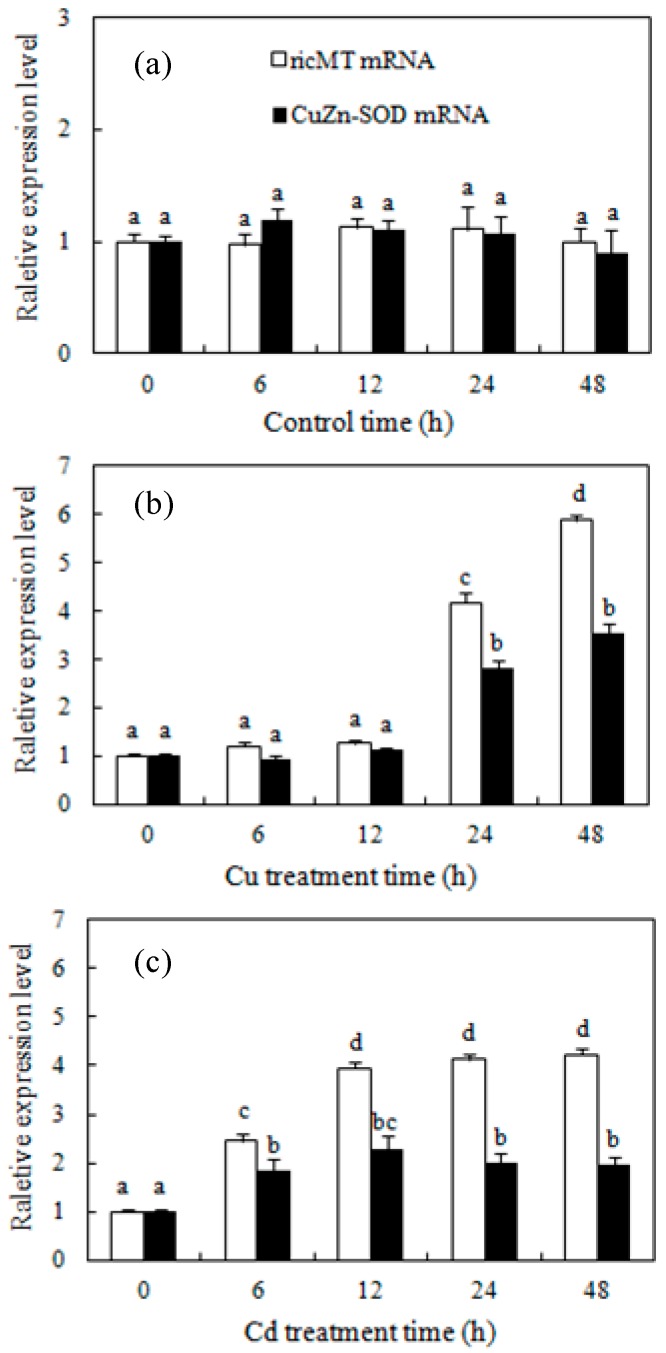
Time course for Cu- and Cd-induced expression of *ricMT* and *CuZn-SOD*. (**a**–**c**) Time course for control (**a**); Cu-induced (**b**) and Cd-induced (**c**) expression of *ricMT* and *CuZn-SOD*. Germinating rice embryos were treated with distilled water (control), 100 μM CuSO_4_ or 100 μM CdCl_2_ for various times (0, 6, 12, 24, and 48 h). Subsequently, radicles were isolated from the germinating seeds for gene analyses by quantitative RT-PCR. Values are means ± SE (*n* = 3) of three separate experiments. Means denoted by the same letter did not significantly differ at *p* < 0.05 according to Duncan’s multiple range test.

The temporal changes in the gene expression of ricMT and CuZn-SOD were analyzed in rice radicles using quantitative RT-PCR. There was no significant difference in the expression level under the control medium within 48 h (Figure 2a). The expression of ricMT and CuZn-SOD mRNA was significantly higher in rice radicles treated with 100 µM Cu or 100 µM Cd for 24 and 48 h than in the control (Figure 2b,c). By contrast, Cd significantly up-regulated the mRNA levels of two proteins under 6 and 12 h treatment, while Cu did not.

### 2.3. ricMT Expression Improved Cu and Cd Tolerance of Rice Suspension Cells

**Figure 3:**
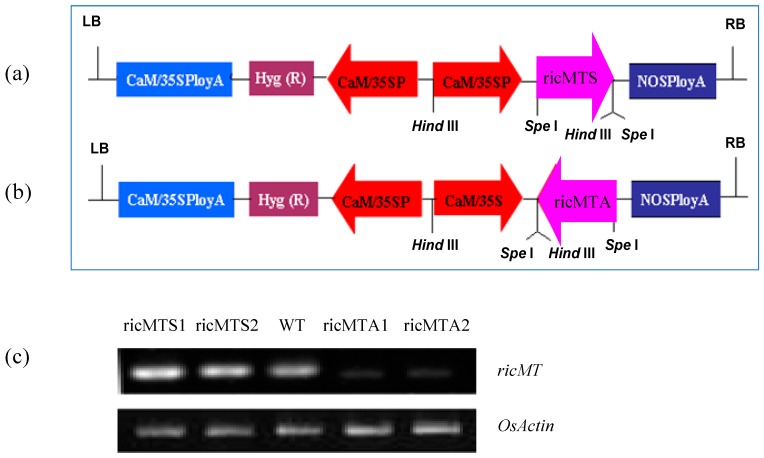
Generation of transgenic rice. (**a**) Diagram of the pCAMBIA1304 vectors harboring sense-*ricMT*; (**b**) Diagram of the pCAMBIA1304 vectors harboring antisense-*ricMT*; (**c**) Semi-quantitative RT-PCR analysis of *ricMT* expression in wild-type and transgenic rice suspension cells. LB represents the left border; RB represents the right border; WT represents the wild-type rice suspension cells; ricMTS1 and ricMTS2 represent two independent sense-*ricMT* transgenic rice suspension cell lines; ricMTA1 and ricMTA2 represent two independent antisense-*ricMT* rice suspension cell lines. Experiments were repeated at least three times with similar results.

To evaluate the roles of ricMT, we generated transgenic rice suspension cells expressing the full-length ricMT cDNA under control of the CaMV 35S promoter using *Agrobacterium* mediated transformation (Figure 3a,b). The expression of *ricMT* in transgenic lines (sense-*ricMT* lines ricMTS1 and ricMTS2, antisense-*ricMT* lines ricMTA1 and ricMTA2) and the wild-type (WT) was analyzed using semi-quantitative RT-PCR (Figure 3c). Rice cells of the sense-*ricMT* lines ricMTS2 and antisense-*ricMT* lines ricMTA2 were used for subsequent rice suspension cell experiments.

**Figure 4:**
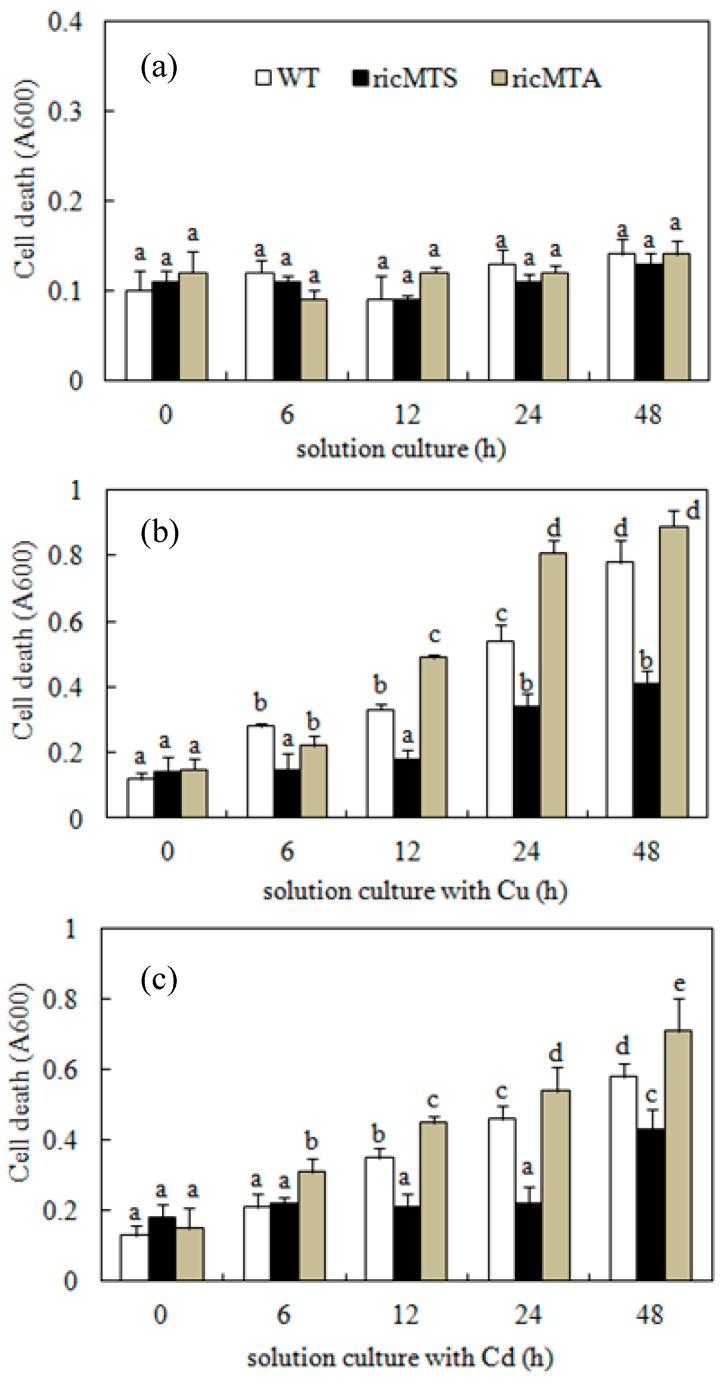
Effect of treatment of Cu and Cd on cell death of rice suspension cells. (**a**–**c**) Rice suspension cells were cultured with control medium (**a**), medium with 100 μM CuSO_4_ (**b**) or medium with 100 μM CdCl_2_ (**c**) for various times (0, 6, 12, 24, and 48 h). Subsequently, aliquots of the suspension cells were stained with Evans blue. Cells were then washed to remove excess stain, ground with a micro-sample pestle in the presence of 0.5% SDS to release trapped stain. The A600 of the supernatant was used to monitor cell death. WT represents the wild-type rice suspension cells; ricMTS and ricMTA respectively represent rice suspension cells of the sense-*ricMT* lines ricMTS2 and antisense-*ricMT* lines ricMTA2. Values are means ± SE (*n* = 3) of three separate experiments. Means denoted by the same letter did not significantly differ at *p* < 0.05 according to Duncan’s multiple range test.

There was no significant difference in the growth rates of WT and transgenic cells in normal medium (Figure 4a). When rice cell cultured on medium supplemented with 100 µM Cu or 100 µM Cd, there was higher cell death rate than that of normal medium. In comparison with the WT, the sense-*ricMT* line (ricMTS2) had a decreased rate of cell death after 6 h Cu treatment or after 12 h Cd treatment; by contrast, the antisense-*ricMT* line (ricMTA2) showed an increased cell death rate at 12 and 24 h of Cu treatment or after 6 h of Cd treatment (Figure 4b,c).

### 2.4. ricMT Expression Decreased H_2_O_2_ Production in Rice Suspension Cells under Cu and Cd Stress

**Figure 5:**
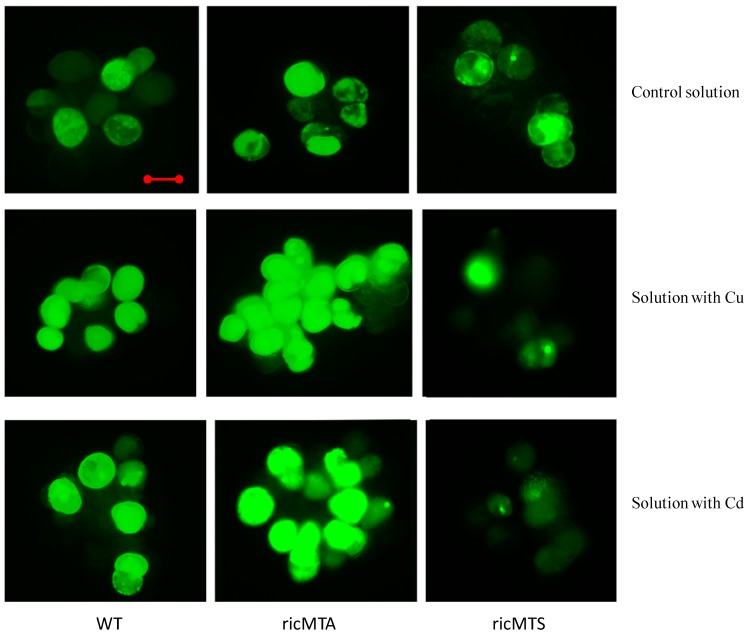
Effect of treatment of Cu or Cd on H_2_O_2_ production of rice suspension cells. Rice suspension cells were cultured with control medium, or medium with 100 μM CuSO_4_ or medium with 100 μM CdCl_2_ for 24 h. Subsequently, the suspension cells were incubated in 20 μM H_2_DCFDA for 20 min. WT represents the wild-type rice suspension cells; ricMTA and ricMTS respectively represent rice suspension cells of the antisense-*ricMT* line and sense-*ricMT* line. Bar, 20 μm. Experiments were repeated at least three times with similar results.

To understand the role of *ricMT* in antioxidant protection, H_2_O_2_ production in rice suspension cells was detected by H_2_DCFDA staining. There was no significant difference in H_2_O_2_ production between WT and transgenic cells in normal medium (Figure 5). In comparison with the control solution, when cultured in medium supplemented with 100 µM Cu or 100 µM Cd for 24 h, the H_2_O_2_ production increased significantly in the rice suspension cells. In comparison with the WT, when cultured in medium supplemented with 100 µM Cu or 100 µM Cd for 24 h, the sense-*ricMT* line (ricMTS2) showed decreased H_2_O_2_ production, while the antisense-*ricMT* line (ricMTA2) showed increased H_2_O_2_ production.

## 3. Discussion

Numerous studies have shown that heavy metals can induce the formation of ROS, including H_2_O_2_, and cause oxidative stress. Cu and Cd toxicity causes an oxidative burst with rapid H_2_O_2_ production and its release into the plant apoplast [27,28,29,30]. In this study, the formation of H_2_O_2_ was observed in Cu- or Cd-treated rice radicles and cells (Figure 1 and Figure 5). Since H_2_O_2_ is relatively stable and can diffuse through cell membranes, it can modulate gene expression and participate in various physiological processes [5,31].

SODs play a key role in the antioxidant defense system through the dismutation of O_2_^•−^ to H_2_O_2_. Excess Cu or Cd treatment increased SOD expression [28,32] and activity [28,29,30], which also influenced the H_2_O_2_ production in plants. It was reported that rice MT transcription was induced in response to both Cu and H_2_O_2_ [18,20]. In this study, the treatment of Cu for 24 and 48 h or Cd for more than 6 h activated the transcription of ricMT and CuZn-SOD in rice radicles (Figure 2). The 6- and 12-h Cu treatments induced significant H_2_O_2_ production, which happened before the expression of *CuZn-SOD* and *ricMT* increased, therefore we guess that Cu-induced H_2_O_2_ production acts upstream from *ricMT* and *CuZn-SOD* expression in the induction of Cu stress. In comparison, the H_2_O_2_ induced by 12-h Cd was lower than the Cu-induced H_2_O_2_, which happened after increased expression of *CuZn-SOD* and *ricMT*, so we guess that it can act downstream from *ricMT* and *CuZn-SOD* expression and block H_2_O_2_ production in rice radicles (Figure 1 and Figure 2). Consistent with our results, the expression of H_2_O_2_-removing enzymes is reported to be up-regulated by excess Cu or elevated endogenous H_2_O_2_ [26,28], and increased H_2_O_2_-removing enzymes decrease H_2_O_2_ production [28,33]. However, overexpressing *CuZn-SOD* also showed increased H_2_O_2_ production in transgenic potato [34]. The co-regulation of *CuZn-SOD* and *MT* expression in yeast may protect against cell toxicity caused by excess Cu [35]. However, the overexpression of *MT* did not protect cultured motor neurons from mutant CuZn-SOD toxicity [36].

In addition to chelating extra metal ions in plant cells via their Cys thiol groups, MTs may also enhance plant tolerance to stress by up-regulating anti-oxidative enzymes to maintain the redox balance and thereby reduce ROS-induced injury [15,17,37,38]. To make clear the role of ricMT on oxidative damage, rice suspension cells of sense- and antisense-*ricMT* transgenic lines were obtained by an *Agrobacterium*-mediated transformation for the first time. In comparison with the WT, the sense-*ricMT* line (ricMTS2) had a decreased rate of cell death after 24 h Cu treatment or 48 h Cd treatment, and the antisense-*ricMT* line (ricMTA2) showed an increased cell death rate after 6 h of Cu treatment or 12 h of Cd treatment (Figure 4b,c). It is reported that MT-overexpressing plants [18,39] or yeast [40,41] have a more efficient antioxidant system with increased enzyme activity against stress conditions. Kumar et al. [11] found that ectopic expression of *OsMT1e-P* protected against oxidative stress primarily through efficient scavenging of ROS.

In the present study, *ricMT* expression blocked the production of H_2_O_2_ in rice suspension cells under Cu or Cd stress (Figure 5). Contrasting the WT, an increase of H_2_O_2_ was accompanied by high cell death rate in antisense-*ricMT* rice lines under Cu and Cd treatment, and a decrease of H_2_O_2_ was accompanied by low cell death rate in sense-*ricMT* lines (Figure 4 and Figure 5). Moreover, Cu, being a redox-active metal, causes higher accumulation of H_2_O_2_ in rice suspension cells than Cd (Figure 5), which is coincident with higher cell death under Cu treatment (Figure 4). Consistent with our results, heterologous expression of *OsMT2c* [18] or *BcMT* [19] in *Arabidopsis* provided increased tolerance against Cu or Cd stress and accumulated lower amounts of H_2_O_2_. In comparison, Cu alone (and not oxidative stress) was reported to induce *MT* expression in *Neurospora crassa* [42].

H_2_O_2_ production in rice radicles was increased before the expression of *ricMT* and *CuZn-SOD* induced by Cu, but not Cd, which suggests that Cu-induced H_2_O_2_ production acts upstream from *ricMT* and *CuZn-SOD* expression in the induction of Cu stress. The overexpression of *ricMT* significantly decreased the rice cell death rate, which was accompanied by lower H_2_O_2_ accumulation in rice cells in response to Cu and Cd stress. This indicates that H_2_O_2_ is involved in the *ricMT*-mediated rice tolerance to Cu and Cd toxicity.

## 4. Materials and Methods

### 4.1. Plant Materials

Rice (*Oryza sativa* L. cv. Wuyunjing No. 7) seeds were surface-sterilized with 5% (*v/v*) sodium hypochlorite (NaClO) for 15 min and washed thoroughly in distilled water. Then, the seeds were germinated on moist filter paper. Twenty seeds were randomly placed on filter paper in 90-mm Petri dishes and germinated in the dark at 25 °C with the distilled water renewed at 2-day intervals. After germinating for 4 days, 5 mL of freshly prepared 100 µM CuSO_4_ solution, 100 µM CdCl_2_ solution, or distilled water (control) was added to the Petri dishes for 0, 6, 12, 24, or 48 h; each treatment was performed in triplicate. The radicles were dissected from germinating rice seeds for quantitative RT-PCR and H_2_O_2_ determination.

### 4.2. Generation of Sense and Antisense ricMT Transgenic Rice

The full-length sequences of sense- and antisense-*ricMT* were obtained by PCR and inserted into the *Spe*I and *Hind*III restriction sites of the plant expression vector pCAMBIA1304 (Cambia, Canberra, Australia) under the control of the cauliflower mosaic virus (CaMV) 35S promoter. Then, pCAMBIA1304 vector harboring the sense- or antisense-*ricMT* was transformed into the rice cultivar Nipponbare by using *Agrobacterium*-mediated transformation [43].

### 4.3. Suspension Cell Cultures

Rice suspension cells were cultured in Chu (N6) medium containing 30 g·L^−1^ of sucrose, 2 mg·L^−1^ of 2,4-d-dicholorophenoxyacetic acid and 0.2 mg·L^−1^ kinetin. After autoclaving, 50 mg·L^−1^ of filter-sterilized hygromycin B was added for positive selection. The cells were maintained in 500-mL Erlenmeyer flasks containing 180 mL of fresh medium, and subcultured every week. For the flask experiments, a 100-mL flask was used, containing 30 mL of fresh medium which was inoculated with 3 g of fresh cells. The cultures were incubated in a gyratory shaking incubator at 28 °C and 120 rpm. For the treatments, cells were used 5 days after subculture, control medium, medium with 100 μM CuSO_4_ or CdCl_2_ were tested at concentrations of 100 µM for 0, 6, 12, 24, or 48 h.

### 4.4. Total RNA Isolation, cDNA Synthesis and Quantitative RT-PCR

Total RNA was extracted using the RNA simple Total RNA Kit (LifeFeng, Shanghai, China) according to the manufacturer’s instructions and then converted to cDNA after DNase I treatment using a PrimeScript^TM^ RT Master Mix (TaKaRa Bio, Tokyo, Japan). Real-time quantitative RT-PCR was performed on a MyiQ Real-Time PCR Detection System (Bio-Rad Hercules, Berkeley, CA, USA) using SYBR Premix Ex Taq (TaKaRa Bio, Tokyo, Japan). The primers for rice CuZn-SOD (AAA33917) mRNA were forward TCATTGGCAGAGCCGTCGTTGT and reverse AGTCCGATGATCCCGCAAGCAA, the primers for ricMT mRNA were forward CACCATGTCGTGCTGGGTGGCAA and reverse CTTCTAGTTGCAGTTGCAGCAGG, and the primers for the internal control *OsActin* were forward TTATGGTTGGGATGGGACA and reverse AGCACGGCTTGAATAGCG. The PCR protocol included an initial 7 min incubation at 95 °C for complete denaturation followed by 40 cycles at 94 °C for 30 s, 60 °C for 30 s, and 72 °C for 30 s. The specificity of the PCR amplification was examined based on a heat dissociation curve (65–95 °C) following the final cycle. Normalized relative expression was calculated using the 2^−ΔΔ*C*t^ (cycle threshold) method.

### 4.5. Hydrogen Peroxide Localization In Situ

The H_2_O_2_ production was detected by the infiltration of H_2_DCFDA, as reported by Ezaki et al. [44] with some modifications. Rice radicles or suspension cells were incubated in 20 μM H_2_DCFDA for 20 min. The excess dye had been removed by washing with distilled water for 1 min, the radicles or suspension cells were transferred to microscope slides and observed with a Zeiss Axio Imager A1 fluorescence microscope (Carl Zeiss, Jena, Germany) fitted with an AxioCam HRc camera to visualize the green fluorescence of the H_2_O_2_-oxidized probe.

### 4.6. H_2_O_2_ Determination in Extracts

The content of H_2_O_2_ in rice radicles from Cu-treated plants was measured by monitoring the A415 of the titanium-peroxide complex following the method described by Jiang et al. [45]. Absorbance values were calibrated to a standard curve established with 0.1–1.0 µM H_2_O_2_.

### 4.7. Evans Blue Assay for Suspension Cell Death

The death of suspension cells was monitored using Evans blue, which is excreted from intact viable cells and is used to estimate cell death spectrophotometrically, as described by Baker and Mock [46]. Briefly, aliquots of treated suspension cells were stained with Evans blue. The cells were washed to remove the excess stain, transferred to 1.5-mL Eppendorf tubes, ground with a micro-sample pestle in the presence of 0.5% SDS to release the trapped stain, and centrifuged to pellet the cellular debris. The A600 of the supernatant was used to quantify cell death.

### 4.8. Statistical Analysis

Data were analyzed using SPSS ver. 16.0 (Statistical Package for Social Science for Windows, SPSS, Inc., Chicago, IL, USA). All values reported in this paper are means ± SE (*n* = 3) of three separate experiments. Means denoted by the same letter did not significantly differ at *p* < 0.05 according to Duncan’s multiple range test.

## Abbreviations

Cys	cysteine
ROS	reactive oxygen species
H_2_DCFDA	2′,7′-dichlorodihydrofluorescein diacetate
MT	metallothionein
SOD	superoxide dismutase
WT	wild-type
